# The potential value of ultrasound in predicting local refractory/relapse events in primary thyroid lymphoma patients

**DOI:** 10.1186/s40644-024-00681-z

**Published:** 2024-03-20

**Authors:** Jiang Ji, Luying Gao, Ruifeng Liu, Xinlong Shi, Liyuan Ma, Aonan Pan, Naishi Li, Chunhao Liu, Xiaoyi Li, Meng Yang, Yu Xia, Yuxin Jiang

**Affiliations:** 1 Department of Ultrasound, Peking Union Medical College Hospital, Chinese Academy of Medical Sciences, Peking Union Medical College, Beijing, China; 2 Department of General Surgery, Peking Union Medical College Hospital, Chinese Academy of Medical Sciences & Peking Union Medical College, Beijing, China; 3 Department of Endocrinology, Peking Union Medical College Hospital, Chinese Academy of Medical Sciences & Peking Union Medical College, Beijing, China; 4 State Key Laboratory of Complex Severe and Rare Diseases, Peking Union Medical College Hospital, Chinese Academy of Medical Sciences and Peking Union Medical College, Beijing, China; 5 Department of Ultrasound, Peking University Shenzhen Hospital, Shenzhen, China

**Keywords:** Primary thyroid lymphoma, Ultrasound, Relapse, Refractory, Treatment outcome

## Abstract

**Background:**

Primary thyroid lymphoma (PTL) is a rare malignant disorder, and ultrasound plays an important role in PTL diagnosis and follow-up surveillance. Prediction of refractory/relapse events in PTL patients is an essential issue, yet no ultrasonic PTL features have been discovered to be related to refractory/local relapse events.

**Methods:**

From January 2008 to September 2022, newly diagnosed PTL patients in our center who underwent standard first-line treatment and received an ultrasound examination before treatment were enrolled. Data regarding patients’ clinical and sonographic features, as well as their therapeutic responses were collected. Subjects with an ideal prognosis were compared to those with refractory/relapse events.

**Results:**

In total, 37 PTL patients were analyzed, including 26 with diffuse large B-cell lymphoma, 2 with follicular lymphoma and 9 with mucosa-associated lymphoid tissue lymphoma. During the median follow-up of 25 months, 30 patients obtained a complete response, 4 were refractory patients, and 3 experienced local relapse. No significant difference was detected in the baseline clinical characteristics between patients with an ideal prognosis and those with refractory/local relapse events. In terms of sonographic features, however, an event-free survival (EFS) curve comparison revealed that patients with bilobar enlargement (defined as an anterior-posterior diameter > 2.5 cm on both sides of thyroid lobes) had a poorer EFS than those without (*P* < 0.0001), and patients with diffuse type had a poorer EFS than those with mixed/nodular types (*P* = 0.043). No significant difference was observed in EFS between patients with or without signs of suspicious cervical lymph node metastasis, rich blood signal distribution or symptoms of trachea compression.

**Conclusions:**

PTL patients with an anterior-posterior diameter > 2.5 cm for both thyroid lobes or PTL patients of the diffuse ultrasound type could be prone to refractory/local relapse events.

**Supplementary Information:**

The online version contains supplementary material available at 10.1186/s40644-024-00681-z.

## Background

Primary thyroid lymphoma (PTL) is a rare but malignant thyroid disorder. The prevalence of PTL in thyroid malignancy is 2–5% and less than 2% in all extranodal lymphomas [1], yet in patients with Hashimoto’s thyroiditis (HT), the prevalence of PTL is elevated [2]. The classification and corresponding treatment of PTL are generally paralleled with that of nodal lymphoma. Hodgkin’s lymphoma and T-cell lymphoma are extremely uncommon in PTL [3], and for B-cell derived non-Hodgkin’s PTL, the most common pathological subtype is diffuse large B-cell lymphoma (DLBCL), followed by mucosa-associated lymphoid tissue lymphoma (MALT) and follicular lymphoma (FL). Regardless of the pathological subtype, the classic clinical manifestation of PTL patients is rapid neck swelling and consequent trachea compression symptoms, such as difficulty breathing [4]. Most PTL patients can achieve a complete response after standard first-line therapy, yet 10–30% of patients relapse after treatment (mostly locally) or are refractory to first-line therapy [5, 6], leading to readmission for treatment. Prediction of refractory/relapse events could promote active surveillance of PTL patients prone to these events and is thus an essential clinical issue. Several studies have discovered factors that are potentially related to relapse/refractory events in PTL patients, but these factors are all clinical features [5, 7–9].

Ultrasound is a useful tool in PTL early screening, diagnostic biopsy and follow-up surveillance. Ota et al. introduced the concept of PTL ultrasound classification according to the sonographic morphology of PTL (diffuse, nodular and mixed type), and this concept has been widely accepted [1]. In addition, various studies have focused on the ultrasonic differentiation of PTL and other thyroid diseases, such as anaplastic thyroid carcinoma, HT, and papillary thyroid carcinoma [9–12]. Some ultrasonic features of thyroid lesions have been reported to be related to relapse or poor prognosis in other thyroid diseases [13, 14]. However, the potential correlation between sonographic features of PTL and PTL patient treatment outcomes, namely, the appearance of relapse/refractory events, has seldom been discussed.

In this study, we reviewed PTL patients with or without local relapse/refractory events in our center, analyzed sonographic features of their thyroid lesions and discovered the potential correlation between PTL ultrasound manifestation and the appearance of relapse/refractory events during follow-up.

## Methods

### Patients

From January 2008 to September 2022, newly diagnosed PTL patients in our center were enrolled. The patient inclusion criteria were as follows: (1) a confirmed diagnosis of PTL and confirmation of the pathological subtype, which was classified according to the 2016 World Health Organization classification standard; (2) underwent a full course of first-line chemotherapy (rituximab plus cyclophosphamide, adriamycin, vincristine and prednisone) and/or radiotherapy according to the pathological subtypes of lymphoma; and (3) received an ultrasound examination before treatment. Patients with a pathological subtype of Hodgkin’s lymphoma, or patients who died before completion of first-line treatment or who were lost to follow-up before completion of first-line treatment were excluded. Patients were followed-up until the failure of first-line therapy, death or transplantation.

### Ultrasonography examinations

**Fig. 1:**
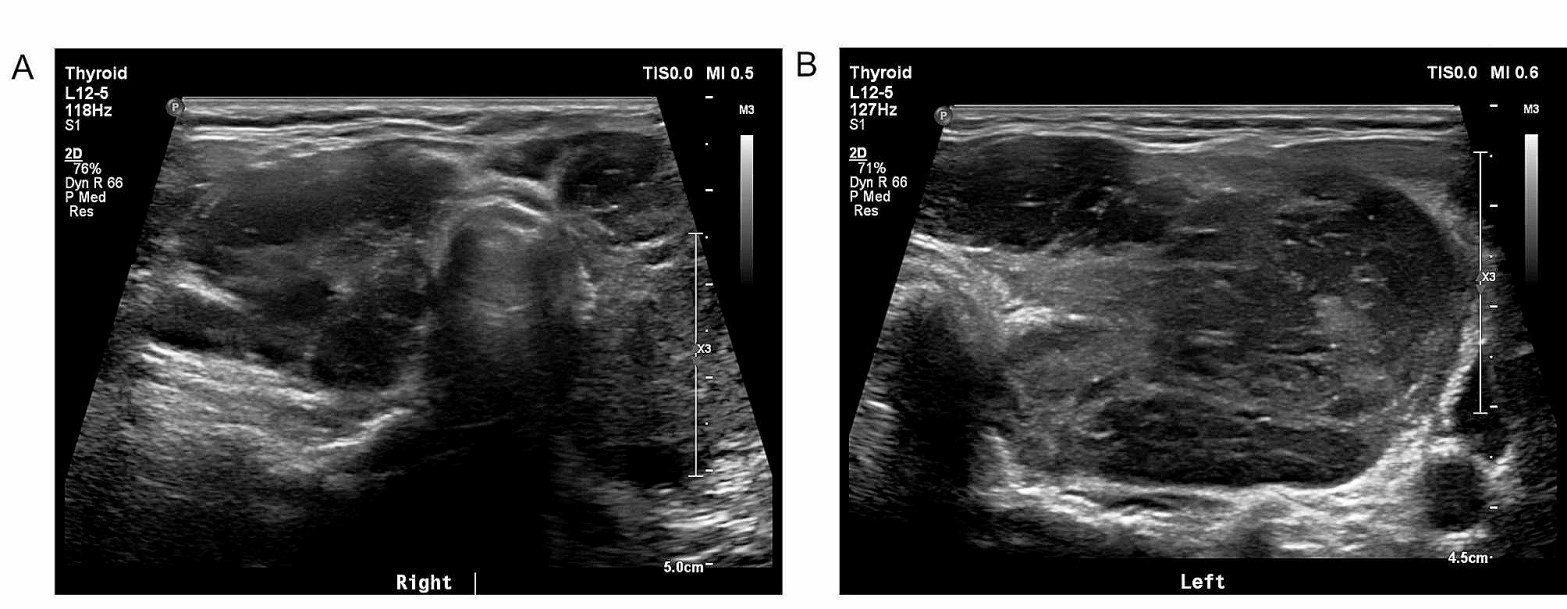
The transverse section of the thyroid from a type-A primary thyroid lymphoma patient. The anterior-posterior diameter was 2.7 cm for the right lobe **(A)**, and 3.2 cm for the left lobe **(B)**. In addition, sonographic features of ultra-hypoecho and strip echo were present for this patient

**Fig. 2:**
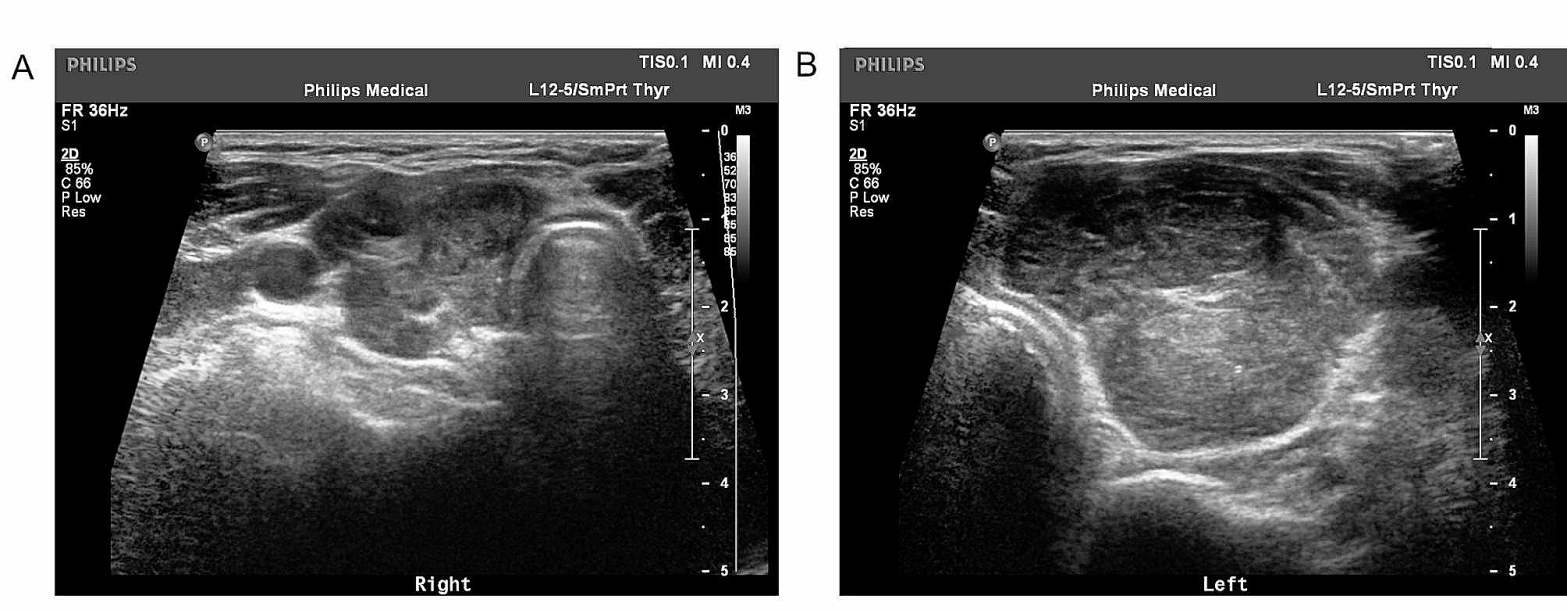
The transverse section of the thyroid from a type-B primary thyroid lymphoma patient. The anterior-posterior diameter was 2.2 cm for the right lobe **(A)**, and 3.4 cm for the left lobe **(B)**. In addition, sonographic features of strip echo and grid echo were present for this patient

A 12 MHz linear probe (GE Logiq E9, US) and a 5–12 MHz linear probe (Philips IU22/EPIQ, the Netherlands) were used to examine patients. The patients underwent multisection ultrasound scans of the thyroid gland and cervical lymph nodes. During examination, the parameters of the ultrasonic apparatus were selected according to the patient condition to obtain an ideal sonograph of the lesion. All ultrasound images were reviewed by two radiologists specializing in ultrasound examination of the thyroid with 5 years of experience. When disagreement occurred between these two radiologists, to obtain a final report, the specific image was reviewed by a third radiologist specializing in ultrasound examination of the thyroid who had 20 years of experience. Sonographic features were extracted from the images, including the ultrasound type of PTL (nodular, diffuse or mixed) determined by Ota et al. [1], the anterior-posterior diameters of both thyroid lobes, the presence of extrahypoecho, strip echo or grid echo, the grade of blood flow signal distribution according to the Adler classification [15], and any suspicious metastasis of cervical lymph nodes showing signs including enlargement in a round shape, disappearance of lymphatic hilum echo, uneven internal echo, and irregular blood flow signals [16]. In this study, the anterior-posterior diameters of both lobes were selected to evaluate the degrees of the thyroid gland enlargement [22], and the patients were classified into three categories accordingly: type A was defined as an anterior-posterior diameter>2.5 cm for both thyroid lobes (Fig. 1); type B was defined as an anterior-posterior diameter>2.5 cm for only one side of the thyroid lobe (Fig. 2); and type C was defined as anterior-posterior diameter ≤ 2.5 cm for both lobes.

### Statistical analysis

Descriptive statistics are presented as frequencies. Continuous variables are presented as the median and range or mean ± SEM. Quantitative data were compared by two-tailed t test or Mann‒Whitney U test, while categorical data were compared using the Chi-square test or Fisher’s exact test. A Kaplan‒Meier curve was used to demonstrate the cumulative event-free survival (EFS) curve, and the curves were compared by the log-rank test. Any variable that was considered clinically or sonographically significant or that showed a univariate correlation with the outcome was included in the multivariate analysis. A two-sided *P* < 0.05 was considered statistically significant. SPSS Statistics (version 25; IBM, Armonk, NY, USA) and GraphPad Prism software (version 5.00; GraphPad Software, San Diego, CA, USA) were used for statistical tests.

## Results

### Patient baseline clinical characteristics

A detailed diagram of patient enrollment is displayed in Supplementary Fig. 1. In total, 37 PTL patients, including 9 males and 28 females, were enrolled in our study, and their clinical characteristics are displayed in Table 1. The median age was 61 (33–84) years. Among the patients, 26 were pathologically classified as DLBCL, 2 as FL and 9 as MALT. During the median follow-up of 25 months, 30 patients obtained an ideal (complete) response, 4 were refractory patients (failed to reach complete response) and 3 experienced local relapse. No significant difference was detected between patients with or without relapse/refractory events in their sex, age, pathological subtype distribution, presence of trachea compression symptoms, presence of HT, presence of lactatedehydrogenase elevation or Ki-67% index. The percentage of IPI low-risk patients was 60.0% for patients with an ideal prognosis and 28.6% for patients with relapse/refractory events, although the difference was not significant (*P* = 0.212).

**Table 1:** Patient clinical baseline characteristics

	Total *N* = 37	Non-relapsed / refractory patients *N* = 30	Relapsed/refractory patients *N* = 7	P value
**Male / n (%)**	9 (24.3%)	8 (26.7%)	1 (14.3%)	0.652
**Age / year, median (range)**	61 (33–84)	61 (33–84)	57 (47–83)	0.885
**Pathological subtype**				
**DLBCL / n**	26	20	6	0.161
**FL / n**	2	1	1	
**MALT / n**	9	9	0	
**IPI low-risk ** **/ n (%)**	20 (54.1%)	18 (60.0%)	2 (28.6%)	0.212
**Presence of trachea compression symptom / n (%)**	22(59.5%)	18 (60.0%)	4 (57.1%)	1.000
**Presence of Hashimoto’s thyroiditis / n (%)**	19 (51.4%)	14 (46.7%)	5 (71.4%)	0.405
**Elevated LDH / n (%)**	22 (59.5%)	18 (60.0%)	4 (57.1%)	1.000
**Ki67 index>30% / n (%)**	29 (78.4%)	22 (73.3%)	7 (100%)	0.308
**Follow-up / month, median (range)**	25 (8-180)	25 (8-151)	37 (8-180)	0.690

DLBCL: diffuse large B-cell lymphoma, FL: follicular lymphoma, MALT: mucosa associated lymphoid tissue lymphoma, IPI: international prognostic index, LDH: lactatedehydrogenase

### Patient sonographic features

**Fig. 3:**
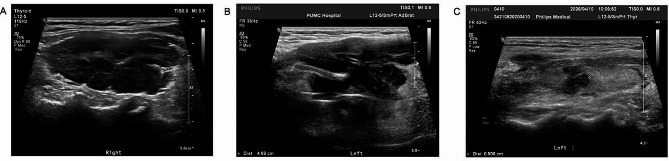
Examples of the diffuse (A), mixed (B) and nodular(C) type of primary thyroid lymphoma, with the left/right lobe of thyroid displayed in the longitude section.

The sonographic features of the enrolled patients are summarized in Table 2. In terms of ultrasound classification, 18 (48.6%) patients were the diffuse type, for which lesions were usually observed in both lobes and had unclear borders (Fig. 3A); 14 (37.8%) were the mixed type (Fig. 3B), for which lesions were patchily distributed in the thyroid gland; and 5 (13.5%) were the nodular type, for which goiter was usually present in one lobe and had a well-defined border (Fig. 3C). No significant difference was detected in the proportion of each ultrasound type between patients with or without relapse/refractory events (*P* = 0.088). For the thyroid enlargement evaluation, the distribution of types A, B and C significantly differed between patients with or without relapse/refractory events (*P* = 0.008). No significant difference was observed in the other evaluated sonographic features, including the presence of grid echo/strip echo/extrahypoecho for the thyroid gland, Adler grade of blood signal in the PTL lesion, and suspicious metastasis of cervical lymph nodes.

**Table 2:**
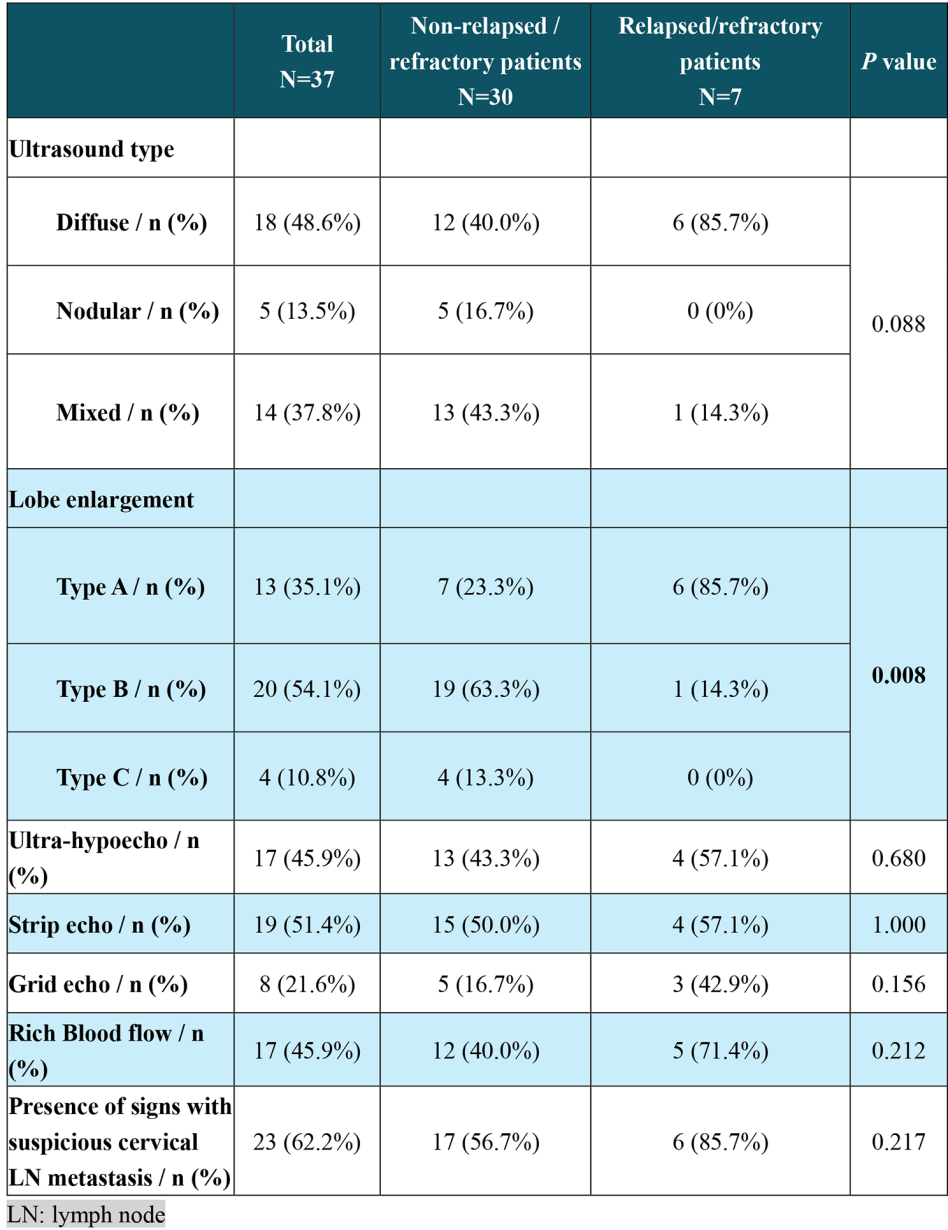
Patient sonographic features

### Follow-up and event-free survival analysis

**Fig. 4:**
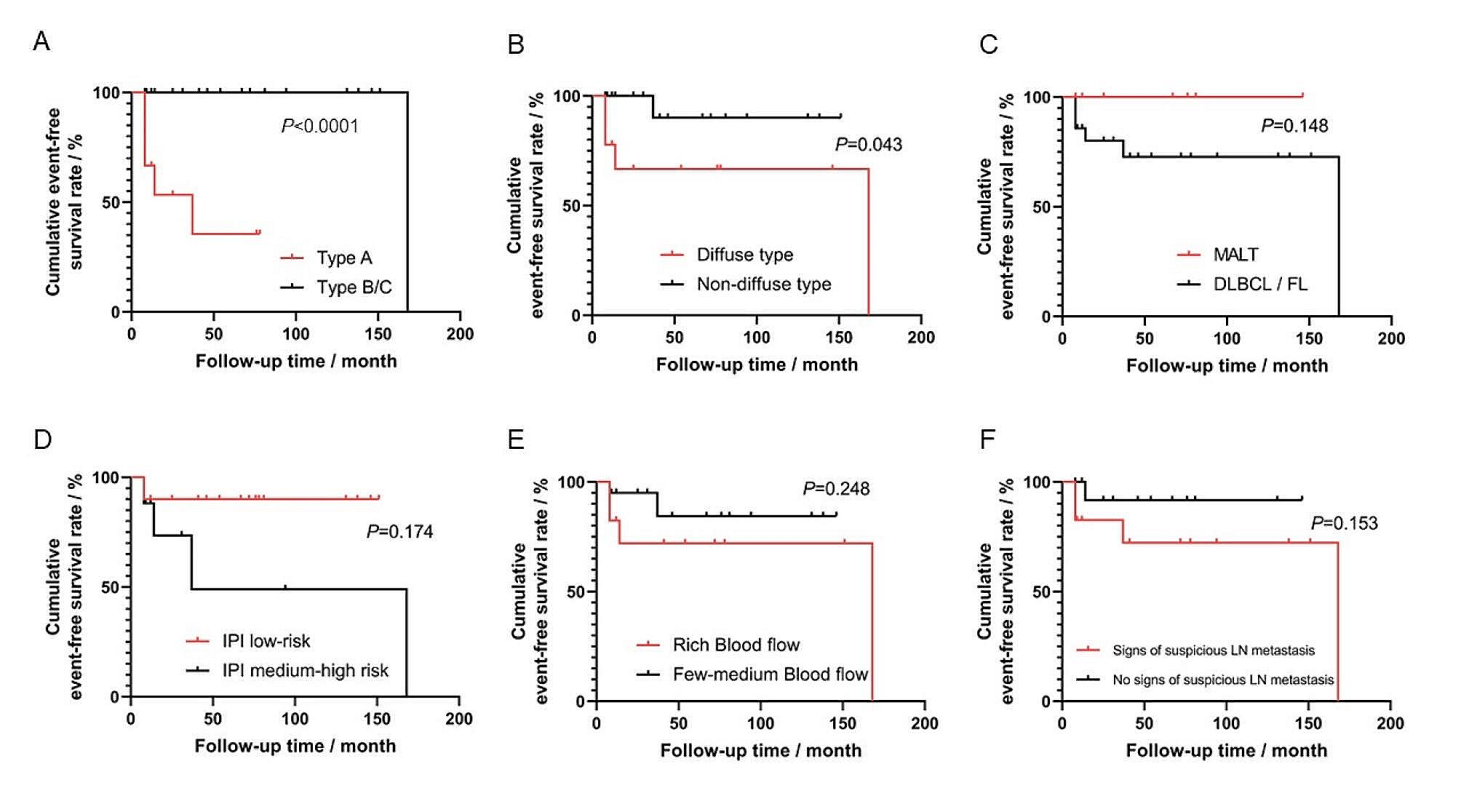
Event-free survival curves of patients with type A of thyroid gland enlargement or type B/C of thyroid gland enlargement **(A)**, diffuse or non-diffuse ultrasound types **(B)**, MALT or other pathological subtypes **(C)**, IPI low-risk or IPI medium-high risk **(D)**, rich or few-medium blood flow **(E)**, or signs of suspicious cervical lymph node (LN) metastasis **(F)**. The differences between two curves in panel A and B were significant.  DLBCL: diffuse large B-cell lymphoma, FL: follicular lymphoma, MALT: mucosa associated lymphoid tissue lymphoma, IPI: international prognostic index

The median follow-up was 25 (8-151) months for patients with an ideal prognosis and 37 (8-180) months for patients with local relapse/refractory events (*P* = 0.690). The follow-up of all patients reached 12 months except for the 4 refractory patients since the follow-up of refractory patients stopped when their first-line therapy was finished. One patient died at the 12th month of follow-up due to pulmonary infection. When defining local relapse/refractory events as events in the analysis of EFS, factors that possibly affected EFS were evaluated, and the results are summarized in Fig. 4. For the types of gland enlargement, a significant difference was found between patients of type A and patients of type B/C in their cumulative EFS curves (*P* < 0.0001, Fig. 4A). In addition, the difference in the EFS curve between patients with diffuse and other ultrasound types was nearly significant (*P* = 0.043, Fig. 4B). A trend of superiority was observed in the EFS of patients with the MALT subtype, who were IPI-low-risk, had few-medium blood flow and had no signs of suspicious LN metastasis, yet the difference was not significant (Fig. 4C-F). Interestingly, no trend of deviation was found between the EFS of patients with or without trachea compression symptoms (*P* = 0.617). However, a multivariate Cox regression analysis of the degree of gland enlargement, ultrasound type, pathological subtype, IPI risk, blood flow and suspicious LN metastasis showed that no factor independently affected the EFS of PTL patients (Supplementary Table 1).

## Discussion

PTL is a rare malignancy, and proper management of PTL patients is essential for a better prognosis, especially for those who relapse after or are refractory to first-line therapy. Early identification of patients who are prone to relapse/refractory events would be helpful in PTL management. As a convenient and widely used approach for PTL evaluation, ultrasound may also play a role in the prediction of PTL treatment outcomes, yet limited studies have addressed this issue. Several studies have discovered factors that are potentially related to relapse/refractory events in PTL patients, but these factors are all clinical features [5, 7, 8]. To our knowledge, we are the first to discover the potential relationship between PTL patients’ ultrasonic features and their treatment outcome.

In our study, the majority of the enrolled PTL patients were female, and over half of the patients were older than 60 years old, which was consistent with most previous studies [1, 17, 18]. The pathological subtypes of the enrolled patients were mainly DLBCL and MALT, and these were also the most common subtypes of PTL [3]. A numerically lower proportion of MALT patients was found for relapsed/refractory PTL patients compared with non-relapsed/refractory patients in our study. Since MALT lymphoma is an indolent pathological subtype of B-cell-derived lymphoma, it is reasonable that PTL patients with the MALT subtype experienced fewer refractory/relapse events. The proportion of IPI low-risk patients was 52.8% in our study, while in Sun et al.’s study, the proportion was 77.5% [18]. The relatively low proportion of IPI-low-risk patients in our study could be attributed to admission rate bias. Although no significant difference was detected, the proportion of IPI-low-risk patients was numerically much higher in patients without relapse/refractory events than in patients with local relapse/refractory events. This difference was reasonable since lymphoma patients with a higher IPI score tended to have a worse EFS [19].

The sonographic features of the enrolled patients were also summarized. In our study, we evaluated the degree of thyroid gland enlargement in the enrolled patients by the anterior-posterior diameter of both lobes. To our knowledge, we are the first to use a quantified definition to describe the enlargement of thyroid lobes in PTL patients and classify thyroid enlargement. The selection of the anterior-posterior diameter of the thyroid gland lobe to describe the degree of gland enlargement was based on the theory that HT would more likely cause an increase in the anterior-posterior diameter instead of the length of the thyroid gland, which is affected more often in hyperthyroidism patients [20]. Since HT would increase the chance of PTL, it would be more reasonable to choose anterior-posterior diameter to evaluate the enlargement of the thyroid gland of PTL patients. A further analysis indicated a significant difference in the proportion of each level of thyroid enlargement between the enrolled PTL patients with or without refractory/relapse events. Over 80% of patients with relapse/refractory events were classified as type A enlargement, which referred to an anterior-posterior diameter > 2.5 cm for both lobes, while for the patients without relapse/refractory events, the proportion of type A was less than 25%. This finding suggested that the degree of thyroid enlargement could be a potential factor for treatment outcome prediction in untreated PTL patients. In addition, most reported sonographic features of PTL were also analyzed in our study [8]. A numerically higher proportion of patients with diffuse type/rich blood flow in thyroid lesions/grid echo in thyroid lesions/suspicious cervical lymph node metastasis was observed for patients with refractory/relapse events, although the difference was not significant. These trends were consistent with previous studies [8, 21]. Compared with the nodular or mixed type, a diffuse pattern of thyroid lesions in PTL patients probably indicated a larger area of lymphoma-affected glands and a more progressive disease pattern.

Based on the findings mentioned above, we conducted a further analysis to discover factors that could affect PTL patients’ EFS. For the degree of thyroid gland enlargement, the EFS of type A patients was significantly poorer than that of type B/C patients. Since a typical clinical presentation of PTL is rapid enlargement of the thyroid mass, the degree of thyroid gland enlargement could represent the severity of disease to some extent. Onal et al. observed a similar phenomenon in which PTL patients with larger tumor sizes had worse relapse-free survival [7]. We also discovered a significant difference between the EFS curves of diffuse-type patients and other types of patients. This finding was consistent with our finding about gland enlargement, as both the degrees of enlargement of thyroid lobes and categorizing the lesions based on diffuse/mixed/nodular types were ways to describe the extent of lymphoma infiltration in the thyroid gland. A trend of better EFS for patients with the MALT subtype, IPI-low-risk, few-medium blood flow and no LN metastasis was also observed, although the EFS curve analysis showed no significant difference. These findings suggested that an anterior-posterior diameter exceeding 2.5 cm for both thyroid lobes and diffuse ultrasound type could be factors that was more sensitive in predicting refractory/relapse events compared with other potential sonographic and clinical factors.

There are some limitations in our study. The sample size was limited due to the low incidence of PTL. All relapsed patients experienced local recurrence of lymphoma instead of distant recurrence, so our findings could not be popularized to both types of relapsed patients. Since data were collected retrospectively from patients’ regular ultrasound examinations before treatment, no data from elastography or other innovative ultrasound technology were included. Nevertheless, our study discovered for the first time that PTL patients whose anterior-posterior diameters of both thyroid lobes exceeded 2.5 cm could be prone to relapse/refractory events, as were PTL patients of the diffuse-ultrasound type. These findings highlighted the role of ultrasound evaluation for PTL patients before treatment since ultrasound is a convenient tool to measure the diameters of the thyroid gland, and diffuse/mixed/nodular ultrasound type is a widely accepted concept in the ultrasonography of PTL. Studies with large sample sizes and ideally prospective studies of PTL patients are required to validate our findings.

## Conclusions

PTL patients with an anterior-posterior diameter > 2.5 cm for both thyroid lobes or diffuse ultrasound type could be prone to refractory/relapse events.

### Electronic supplementary material

Below is the link to the electronic supplementary material.

Supplementary Material 1

Supplementary Material 2

Supplementary Material 3

Supplementary Material 4

## Data Availability

The datasets of the current study are available from the corresponding author upon reasonable request.

## Abbreviations

DLBCL: diffuse large B-cell lymphoma
EFS: event-free survival
FL: follicular lymphoma
HT: Hashimoto’s thyroiditis
IPI: international prognostic index
MALT: mucosa-associated lymphoid tissue lymphoma
PTL: primary thyroid lymphoma
