# Supplementary material for: The potential value of ultrasound in predicting local refractory/relapse events in primary thyroid lymphoma patients
Source: Cancer Imaging. 2024 Mar 20;24:39. doi: 10.1186/s40644-024-00681-z (PMC10953231; doi:10.1186/s40644-024-00681-z)
Supplement: Supplementary file 1 — Supplementary Material 1 [file 40644_2024_681_MOESM1_ESM.pdf]

Editing Certificate

This document certifies that the manuscript

The potential value of ultrasound in predicting local refractory/relapse events for primary thyroid lymphoma patients

prepared by the authors

Jiang Ji*, Luying Gao*, Ruifeng Liu, Xinlong Shi, Liyuan Ma, Aonan Pan, Naishi Li, Chunhao Liu, Xiaoyi Li, Meng Yang, Yu Xia&, Yuxin Jiang&

was edited for proper English language, grammar, punctuation, spelling, and overall style by one or more of the highly qualified native English speaking editors at AJE.

This certificate was issued on **October 10, 2023** and may be verified on the AJE website using the verification code **CB2A-BF99-53FB-E185-A7BA** .

Neither the research content nor the authors' intentions were altered in any way during the editing process. Documents receiving this certification should be English-ready for publication; however, the author has the ability to accept or reject our suggestions and changes. To verify the final AJE edited version, please visit our verification page at aje.com/certificate. If you have any questions or concerns about this edited document, please contact AJE at support@aje.com.

AJE provides a range of editing, translation, and manuscript services for researchers and publishers around the world. For more information about our company, services, and partner discounts, please visit aje.com.
