# Supplementary material for: The potential value of ultrasound in predicting local refractory/relapse events in primary thyroid lymphoma patients
Source: Cancer Imaging. 2024 Mar 20;24:39. doi: 10.1186/s40644-024-00681-z (PMC10953231; doi:10.1186/s40644-024-00681-z)
Supplement: Supplementary file 4 — Supplementary Material 4 [file 40644_2024_681_MOESM4_ESM.docx]

**Supplementary Table 1** Multivariate Cox regression analysis for event-free survival (EFS)

| **Factors** | ***P* value** |
| --- | --- |
| **Type A of thyroid gland enlargement** | 0.649 |
| **Diffuse type** | 0.514 |
| **Rich blood flow** | 0.541 |
| **Signs of suspicious cervical LN metastasis** | 0.378 |
| **MALT subtype** | 0.703 |
| **IPI low-risk** | 0.541 |

MALT: mucosa associated lymphoid tissue lymphoma, IPI: international prognostic index, LN： lymph node

**
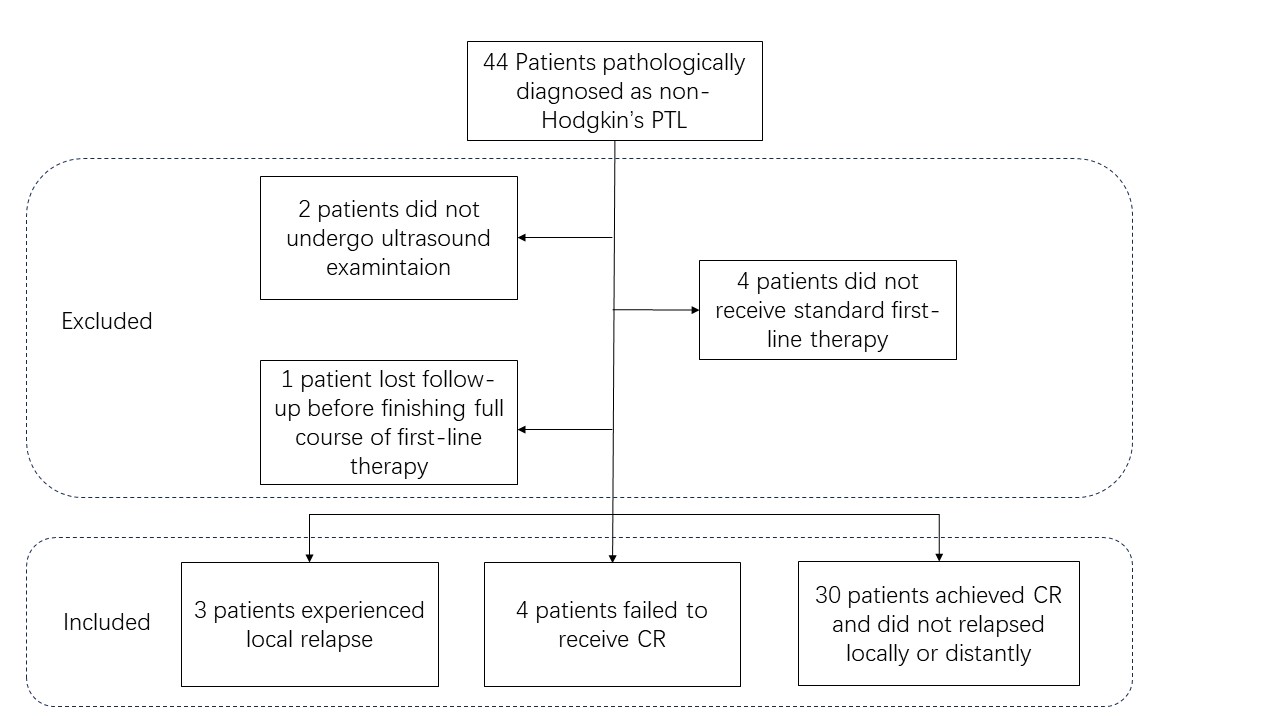
**

**Supplementary Figure 1** The detailed diagram of patient enrollment.
