# Supplementary material for: The potential value of ultrasound in predicting local refractory/relapse events in primary thyroid lymphoma patients
Source: Cancer Imaging. 2024 Mar 20;24:39. doi: 10.1186/s40644-024-00681-z (PMC10953231; doi:10.1186/s40644-024-00681-z)
Supplement: Supplementary file 3 — Supplementary Material 3 [file 40644_2024_681_MOESM3_ESM.docx]

Title: The potential value of ultrasound in predicting local refractory/relapse events in primary thyroid lymphoma patients

Dear Editors and Reviewers,

Thank you for your advice and comments on our manuscript: The potential value of ultrasound in predicting local refractory/relapse events in primary thyroid lymphoma patients. According to your comments, we have made significant modifications to our manuscript to ensure that our article is more accurate and convincing. We have provided a revised manuscript with the changes in response to the reviewers’ comment. The detailed point-by-point responses are listed as follows:

Reviewer reports:

Section Editor: Please have the paper reviewed by English editing services or a native English speaker and submit a tracked version as a supplementary file

**Response: The manuscript has been polished, and a tracked version has been uploaded as a supplementary file.**

Reviewer #1: Comments

1. Method/patient - In general, since relapse in primary thyroid lymphoma typically occurs years later, it would be helpful to either follow up for at least one year or align the follow-up period to provide accurate information.

**Response: The follow-up of all patients reached 1 year except for the 4 refractory patients, since the follow-up of refractory patients stopped when their first-line therapy was finished. Nevertheless the follow-up was comparable between patients with an ideal prognosis and patients with local relapse/refractory events (37 (8-180) *vs.* 5 (8-151) months, *P*=0.690).**

2. Abstract - All abbreviations should have their full names written at the beginning. This should be applied to both the abstract and the main text. Please also use the full name for "EFS" in the abstract.

**Response: The full names of abbreviations have been added both in the abstract and in the main text.**

3. Result - Local recurrence and distant recurrence have different timeframes, patterns, and prognoses. Although this manuscript did not classify them separately as relapse, it is believed that classification may be necessary for the reasons mentioned above.

**Response: All the relapsed patients experienced local recurrence. This information has been clarified in the manuscript.**

4. Figure 2 - What is the meaning of Figure 2 is intend to in these manuscript?

**Response: The rapid swelling of thyroid gland and a consequent trachea compression symptom are the classic clinical manifestations of primary thyroid lymphoma, so we wondered if there was correlation between the severity of trachea compression symptom and local relapse/refractory events. Interestingly, the event-free survival curves of patients with or without trachea compression symptom were quite similar. We intended to use the original Figure 2 to emphasize that compared with clinical symptoms, some ultrasonographic features could be more sensitive in detecting the local relapse/refractory events. Since our intention was not clearly displayed, the original Figure 2 was removed from the manuscript.**

Reviewer #2: The manuscript describes interesting research of ultrasound in predicting refractory/relapse events for primary thyroid lymphoma patients and discovers the potential relationship between PTL patients' ultrasonic features and their treatment outcome. However the number of PTL in your center is relatively small, with only 36 cases in nearly 15 years. Can you obtain more cases to support the results and conclusions?

**Response: We carefully searched for suitable PTL patients in our database again, and found another patient meeting the inclusion criteria. Now 37 patients were included in our study. Since some of the PTL patients in our center did not receive a standard first-line therapy due to personal reasons, these patients were excluded from the study, which further reduced the number of included patients.**

1. Suggest a detailed diagram of patient enrollment in the method section.

**Response: The diagram has been added as below (Supplementary Figure 1).**

2. P6 Line20-22："To evaluated if there was enlargement of the thyroid gland"
   Do you have any references or how do you define them?

**Response: The enlargement of thyroid gland was defined in this study as anterior-posterior diameter >2.5cm.**

3. P6 Line18-20 any suspicious metastasis of cervical lymph nodes showing signs including enlargement in a round shape, disappearance of lymphatic hilum echo, uneven internal echo, and irregular blood flow signals. Please cite relevant reference.

**Response: The reference has been cited in the manuscript as follows:**

**Jain NK, Mostoufi-Moab S, Hawkes CP, Nelson ND, Surrey LF, Jones ZS, et al. Extrathyroidal Extension is an Important Predictor of Regional Lymph Node Metastasis in Pediatric Differentiated Thyroid Cancer. Thyroid. 2020;30(7):1037-43.**

4.There is too little ultrasound features in the table 2, such as calcification, but the discussion section (P12 Line1-2) mentioned it.

**Response: As was discussed in the manuscript, there are some ultrasound features that worth analyzing in other malignant thyroid diseases such as carcinoma or anaplastic thyroid cancer, yet the ultrasound manifestation of primary thyroid lymphoma is distinct from the malignancies mentioned above, so they were not included in the Table 2.**

5. The result part didn't show any ultrasound images.

**Response: Ultrasound images have been added as follows (Figure 1-3):**

**Figure 1** The transverse section of the thyroid from a type-A primary thyroid lymphoma patient. The anterior-posterior diameter was 2.7cm for the right lobe (A), and 3.2cm for the left lobe (B). In addition, sonographic features of ultra-hypoecho and strip echo were present for this patient.

**Figure 2** The transverse section of the thyroid from a type-B primary thyroid lymphoma patient. The anterior-posterior diameter was 2.2cm for the right lobe (A), and 3.4cm for the left lobe (B). In addition, sonographic features of strip echo and grid echo were present for this patient.

**Figure 3** Examples of diffuse (A), mixed (B) and nodular (C) type of primary thyroid lymphoma.

6. P11 line 19-22 it's not suitable to compare CT pattern with ultrasound pattern. The discussion part should add more content.

**Response: The discussion of CT pattern has been removed. More content has been added in the discussion part as follows:**

**The selection of anterior-posterior diameter of thyroid gland lobe to describe the degree of gland enlargement was based on the theory that Hashimoto’s thyroiditis (HT) would more likely cause the increase of anterior-posterior diameter instead of the length of thyroid gland, which is increased more often in hyperthyroidism patients.** **Since the incidence of PTL is higher in HT patients, it would be more reasonable to choose anterior-posterior diameter to evaluated the enlargement of thyroid gland of PTL patients.**

7. Why you only choose EFS, but no OS, please explain.

**Response: In our study, only one patient died in the follow-up, which made analysis of OS less practicable.**

8.In discussion part, the limitation of this study was not mentioned.

**Response: The limitation of this study has been added in the discussion part as follows:**

**There are some limitations in our study. The sample size was limited due to the low incidence of PTL. All relapsed patients experienced local recurrence of thyroid instead of distant recurrence, so our findings could not be popularized to both types of relapsed patients. Since data were collected retrospectively from patients’ regular ultrasound examinations before treatment, no data of elastography or other innovative ultrasound technology were included.**

9.The whole passage needs to be polished by native English speakers.

**Response: The manuscript has been polished, and a tracked version has been uploaded as a supplementary file.**
